# Impact of COVID-19 on ischemic stroke care in Hungary

**DOI:** 10.1007/s11357-021-00424-z

**Published:** 2021-08-18

**Authors:** Péter Pál Böjti, Géza Szilágyi, Balázs Dobi, Rita Stang, István Szikora, Balázs Kis, Ákos Kornfeld, Csaba Óváry, Lóránd Erőss, Péter Banczerowski, Wojciech Kuczyński, Dániel Bereczki

**Affiliations:** 1 János Szentágothai Doctoral School of Neurosciences, Semmelweis University, Balassa utca 6., Budapest, 1083 Hungary; 2 National Institute of Mental Health, Neurology and Neurosurgery, Budapest, Hungary; 3 Department of Neurology, Medical Centre, Hungarian Defence Forces, Budapest, Hungary; 4 Department of Probability Theory and Statistics, Eötvös Loránd University, Budapest, Hungary; 5 Neuroepidemiology Research Group ELKH, MTA-SE, Budapest, Hungary; 6 Department of Neurosurgery, Semmelweis University, Budapest, Hungary; 7 Department of Sleep Medicine and Metabolic Disorders, Medical University of Lodz, Lodz, Poland; 8 Department of Neurology, Semmelweis University, Budapest, Hungary; 9 European Academy of Neurology, EANcore COVID-19 Task Force, Vienna, Austria

**Keywords:** COVID-19, Hungary, Stroke, Thrombolysis, Thrombectomy, Epidemiology

## Abstract

Data about the coronavirus disease 2019 (COVID-19) pandemic’s collateral damage on ischemic stroke (IS) care during the second epidemic wave in Central Europe are limited. We sought to evaluate the impact of the COVID-19 outbreak on Hungarian IS care during the two epidemic waves. This retrospective observational study was based on a nationwide reimbursement database that encompasses all IS admissions and all reperfusion interventions, i.e., intravenous thrombolysis (IVT) and endovascular therapy (EVT) from 2 January 2017 to 31 December 2020 in Hungary. COVID-19 pandemic’s effect on the number of IS admissions and reperfusion interventions were analyzed using different statistics: means, medians, trends, relative rates, and linear relationships. The mean and median values of IS admissions and reperfusion interventions decreased only in some measure during the COVID-periods. However, trend analysis demonstrated a significant decline from the trends. The decline’s dynamic and amplitude have differed for each variable. In contrast to IVT, the number of IS admissions and EVTs negatively correlated with the epidemic waves’ amplitude. Besides, the decrease in the number of IS admissions was more pronounced than the decrease in the number of reperfusion interventions. Our study demonstrated a significant disruption in IS care during the COVID-19 epidemic in Hungary, in which multiple different factors might play a role. The disproportionate reduction of IS admission numbers could partially be explained by the effect of health emergency operative measures and changes in patients’ social behavior. Further studies are needed to evaluate the causes of our observations.

**Supplementary Information:**

The online version contains supplementary material available at 10.1007/s11357-021-00424-z.

## Introduction

Cerebrovascular diseases are the second leading cause of death worldwide. Up to 50% of stroke survivors are chronically disabled, which causes a tremendous public health burden with severe economic and social consequences [1]. Over the past decade, the treatment of acute ischemic stroke (IS) has undergone fundamental changes due to the high-quality evidence that shows reperfusion interventions (intravenous thrombolysis, mechanical thrombectomy) within the 4.5 or 6 h of stroke onset can reduce the risk of death or disability and improve functional outcome. In recent years, the range of acute IS patients eligible for reperfusion interventions has further expanded as studies showed that these treatments could be used effectively up to 24 h after symptom onset in certain cases [2–9].

Coronavirus disease 2019 (COVID-19) pandemic reached Hungary on 4 March 2020, and by the end of May 2021, with the 83 thousand cumulative COVID-19 cases per million people, Hungary was one of the most severely affected countries in Europe and the European Union. Considering the COVID-19 outbreak’s extent in neighboring countries, Hungary is in the middle of the range in the Central European region. The impact of the ongoing epidemic on the Hungarian population is further emphasized by the particularly high number of COVID-19 deaths (3000 deaths per million people by the end of May 2021). It is important to note that testing capacities and case definitions influence the number of COVID-19 cases and COVID-19 related deaths. Therefore further adjustment might be needed for accurate comparison between countries [10–13].

With a wide range of variations, studies from all countries reported some negative impact of the COVID-19 pandemic on IS care [14–36]. Although some centers did not observe changes in the number of IS admissions, the vast majority of studies showed a remarkable decrease, with or without a significant decline in the number of reperfusion interventions [14, 18–20, 22–24, 26–28, 31–33, 35]. Data about the impact of the COVID-19 pandemic on IS care of Central Europe are limited. To date, two regional studies from Poland and Hungary demonstrated a marked reduction in the number of IS admissions and reperfusion treatments during the first wave of the SARS-CoV-2 (severe acute respiratory syndrome coronavirus 2) epidemic [17, 30]. The effect of the second wave of the COVID-19 pandemic on IS care is unknown in the current literature. In Hungary, the second wave of the COVID-19 outbreak was different from the first wave: the number of SARS-CoV-2 related infections and deaths were substantially higher, pressure on the healthcare system was more intense, while the confinement measures were considerably milder [10, 12].

We sought to evaluate and quantify IS care dynamics by analyzing the number of IS admissions and reperfusion interventions during the first two waves of the COVID-19 pandemic in Hungary by comparison to baseline and control periods.

## Methods

### Data source

This study was based on the reimbursement database of the National Health Insurance Fund of Hungary (NHIFH). The NHIFH database prospectively registers all healthcare activities performed by healthcare providers supervised by the National Healthcare Service Center of Hungary (NHSC). Hungary has a single-payer healthcare financing system, and NHSC is the largest supporter of healthcare services in Hungary, serving 9.8 million people [37, 38]. In summary, our database encompasses all admissions for IS and all reperfusion interventions — intravenous thrombolysis (IVT) and endovascular therapy (EVT) — performed by healthcare providers supervised by NHSC from 2 January 2017 to 31 December 2020. All patient data were obtained in an anonymized form from the NHIFH.

We used the ICD-10 (10th version of the International Statistical Classification of Diseases and Related Health) I63, I64, and I66 codes to evaluate the number of IS admissions from the reimbursement database of NHIFH. A recent study showed that the cerebrovascular ICD-10 codes submitted for reimbursement purposes in Hungary could be used reliably for stroke epidemiological studies [39]. Since some institutes use the ICD-10 I66 code instead of the I63 code, we used this code in addition to I63 and I64 codes to evaluate the number of IS admissions. However, this method could result in an overestimation of IS incidence with an 8% maximal value, based on NHSC calculation [40]. Some authors suggest using not only the main discharge diagnosis but diagnoses in all five positions (i.e., main diagnosis for admission; basic disease; accompanying disorder; complication; cause of death) to select IS patients in an administrative database with maximal sensitivity [39, 41]. We computed the number of IS admissions as the number of cases where ICD I63 or I64 or I66 codes presented in any of these five discharge diagnosis positions. With this approach, in contrast to stroke mimics (conditions not resulting from cerebral ischemia that present with neurological symptoms indistinguishable from a stroke), the following group of patients could be included in the cohort: acute ISs (patients admitted with acute onset neurological symptoms caused by cerebral ischemia), non-acute ISs (patients formerly treated with acute IS admitted for follow-up investigations), in-hospital ISs (patients admitted for non-stroke reasons but had an IS while hospitalized), IS chameleons (patients presented with clinical symptoms suggestive of another condition, which represents IS), and incidental asymptomatic cerebral infarcts (patients who were hospitalized for a condition other than stroke and had a brain imaging that showed an incidental asymptomatic brain infarct). Each patient counted only at the time of admission in a given week. However, early readmissions (discharged IS patients who were readmitted within a short time interval) were captured from the NHIFH database as separate IS cases, which could result in an overestimation of IS incidence.

To compute the number of IVTs and EVTs, we used the Orvosi Eljárások Nemzetközi Osztályozása (OENO) and the Homogén Betegségcsoportok (HBCs) codes, which are the Hungarian adaptations of International Classification of Procedures in Medicine codes, and Diagnosis Related Groups [42, 43].

IVT has clinical indications other than neurological ones, but acute IS is the only condition where IVT is performed in neurology. Thus, using the OENO code of IVT (OENO 06042), we first identified all IVT cases, irrespectively of the clinical indication. Then, we excluded the non-neurological cases by excluding cases where the HBCs showed other than a neurological indication. IVT cases where HBCs code was missing were included in the analysis, which could overestimate the number of IVTs, but do not alter our goal to detect changes in a process. With this approach, IVT performed in stroke mimics and in-hospital ISs might be included in the cohort.

For EVT coding, most Hungarian neurointerventional facilities use (Type I coding) the OENO 33933 code (intracranial transarterial revascularization therapy). However, two neurointerventional institutions use the OENO 53958 code (intracranial percutaneous transluminal angioplasty) in part or in full instead of the 33933 code (Type II coding) [44]. Therefore we used both codes to obtain the best estimate of EVT numbers.

Our analysis of the Hungarian SARS-CoV-2 data was based on the Our World in Data GitHub database (sourced from the COVID-19 Data Repository by the Center for Systems Science and Engineering at Johns Hopkins University) [12].

### Study periods

The first SARS-CoV-2 infection occurred in Hungary on 4 March 2020 (10th week of 2020). On 11 March 2020 (11th week of 2020), the Hungarian government declared a state of emergency, which lasted until 18 June 2020 (25th week of 2020). With a repeated surge of COVID-19 cases, on 1 September 2020 (36th week of 2020), a COVID-19 entry control scheme was instituted, and on 4 November 2020 (45th week of 2020), a state of emergency went into effect again. Up to date, containment measures are still in place [10–12, 45].

**Fig. 1:**
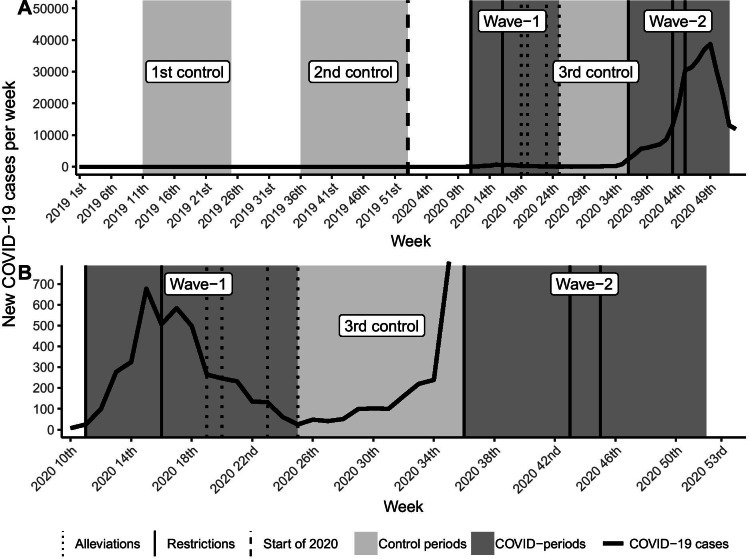
Timeline and summary of the study periods. These graphs summarize the study periods and present them in a timeline, illustrating their temporal relationship to the COVID-19 epidemic waves in Hungary. Dates of the most important restrictive and alleviative health emergency operative measures are also marked in the timelines. (**a**) illustrates both COVID-19 and control periods, while (**b**) focuses on the COVID-periods. Wave-1 period: 11th–25th weeks of 2020. Wave-2 period: 36th–52nd weeks of 2020. 1st control period: 11st–25th weeks of 2019. 2nd control period: 36th–52nd week of 2019. 3rd control period: 26th–35th weeks of 2020. COVID-19, coronavirus disease 2019; COVID-periods, periods of coronavirus disease 2019

The study periods are summarized in Fig. 1. Based on the epidemic’s dynamic, we defined two COVID-periods (Wave-1 and Wave-2) as representative of the first and the second waves of the COVID-19 epidemic in Hungary. Wave-1 period was defined as a 15-week long interval between the 11th and 25th weeks of 2020. Wave-2 period was defined as a 17-week long period between the 36th and 52nd weeks of 2020. The 10-week long interval between the Wave-1 and Wave-2 period designated an epidemic interlude (3rd control period, 26th–35th weeks of 2020). Data of the COVID-periods were compared to their respective periods of 2019 (1st control period: 11th–25th weeks of 2019; 2nd control period: 36th–52nd week of 2019) and with the epidemic interlude. The comprehensive interval of the 11th–53rd weeks of 2020, which extends from the start of the Wave-1 period to the end of 2020, was used in the analyses to study the relationship between the number of COVID-19 cases and the investigated variables. Data from the 1st week of 2017 to the 10th week of 2020 were defined as a baseline period for trend analysis.

Dates of the most important restrictive and alleviative health emergency operative measures were marked and used during the visual-statistical analyses (Fig. 1, 3,4, 5): 11th week of 2020, order of complete restriction of elective health care; 16th week of 2020, order to make 50–60% of beds free to COVID-care; 19th, 20th, 23rd, 25th weeks of 2020, four-step alleviation of restrictive health measures; 36th week, order to make 20% of beds free to COVID-care; 43rd week of 2020, orders to significantly expand the COVID-designated hospitals’ number and make 20–30% of beds free to COVID-care; 45th week of 2020, orders to make 40% of beds free to COVID-care, to designate almost every hospital for COVID-care, and to partially restrict elective health care [12, 46–54].

### Statistical analysis

Statistical analysis was conducted on all characteristics (IS admissions, IVTs, EVTs) both separately and together. The smallest timeframe considered was weekly patient numbers due to the nature of the data.

We analyzed the COVID-19 epidemic waves’ effect on the patient numbers with different tools: means, medians, trends, relative rates, and linear regression. The mean and median differences were tested with *t* test and Wilcoxon-Mann–Whitney test, respectively. Differences between the COVID-periods and their respective controls were compared with the paired version of the tests. However, the COVID-periods and the epidemic interlude were compared with non-paired tests due to differing lengths. Since similar findings were apparent using both means and medians, median-based data are presented as an online supplementary material (Table S1).

Trends and unexpected changes in patient numbers were analyzed using control charts, which are simple visual-statistical tools for detecting changes in a process and are widely used in outbreak analysis [55–58]. The basic idea was to analyze a baseline timeframe (1st week of 2017–10th week of 2020) where it can be assumed that everything is in order and set up definitions for normal behavior. All data were linearly de-trended and standardized; thus we obtained z-scores. The potential effect of heteroscedasticity and seasonality was considered. However, we ultimately decided not to transform the data further due to two reasons: one was not to “over standardize” the data (i.e., categorize possibly extreme behavior during baseline as normal). The second was that these effects could be easily detected and taken into account visually. The z-cores were put on control charts, and the 2 and 3 standard deviation (SD) control limits were set. Changes in z-scores were then determined using Western Electric rules [56]. We also conducted statistical testing on the z-scores because compared to the raw numbers’ means and medians; z-scores consider the trends based on the whole study period.

The rate of IVTs or EVTs relative to the number of IS admissions was also analyzed using control charts. These control charts used the same methodology as described above, but the de-trended and standardized IVT or EVT numbers were first divided by the number of IS admissions to get the relative number of patients.

Linear regression was used to analyze the relationship between the new or cumulative COVID-19 cases per week in Hungary and the weekly number of IS admissions, IVTs, or EVTs. The linear regression took the number of COVID-19 cases per week as the explanatory variable and the weekly IS admission, IVT, or EVT numbers as the outcome one.

While statistical tests did not use the incomplete last week of 2020 (data only for the first 4 days of the week were available), it was included in linear regression analysis and control charts to make the analysis and visual assessment as complete as possible. In this regard, when the characteristics were analyzed by themselves, the last patient number was multiplied by 7/4 to boost it to a whole week level, but when the IVT and EVT numbers were divided by the number of IS admissions, the ratio was left because both data were equally incomplete; thus their ratio is valid.

Due to the different OENO coding practices for EVT, a correction had to be implemented to obtain the best estimate of patient numbers. This correction was based on the Type I coding centers by dividing the number of 53958 codes by the sum of the number of 33933 and 53958 codes, taking into account the whole study period. This gave an estimate of the true ratio of the 53958 codes, which was used to adjust the number of procedures in the Type II coding centers by multiplication. In the end, we added these adjusted 53958 numbers to the 33933 numbers.

R version 4.0.3 was used for data analysis with packages forecast, rgdal, ggplot2, ggpubr, gridExtra, flextable, and tableone.

### Ethics

This study was approved by the Institutional Review Boards/Institutional Ethics Committee of the National Institute of Clinical Neurosciences (predecessor institution of the National Mental, Neurological and Neurosurgical Institute; approval number: IKEB 2/2021) and by the Scientific and Research Ethics Committee of the Health Sciences Council of Hungary (ETT-TUKEB, approval number: IV/678–1/2021/EKU). Written informed consent was waived due to the non-interventional, anonymized, and retrospective character of the investigation.

## Results

**Fig. 2:**
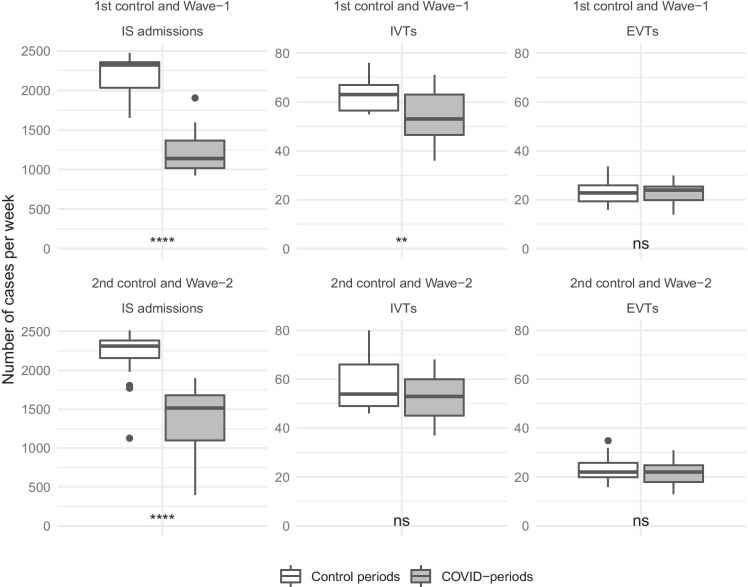
Changes in the raw weekly number of IS admissions and reperfusion interventions during the COVID-periods. This figure shows the raw weekly number of IS admissions, IVTs, and EVTs in the COVID-periods and their respective controls using standard box plots. *p* values of the paired Wilcoxon-Man-Whitney tests, which compare the COVID-periods to their respective controls, are also presented. full dots: Tukey-defined outliers; *p* value: ns (not significant) *p* > 0.05, **p < 0.01, ****p < 0.0001; IS, ischemic stroke; IVT, intravenous thrombolysis; EVT, endovascular therapy; COVID-periods, periods of coronavirus disease 2019

**Fig. 3:**
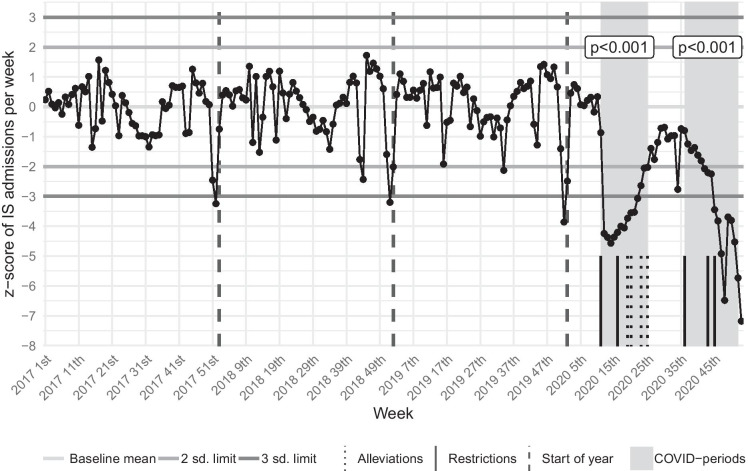
Control chart of IS admissions. This graph visualizes the trend and changes using the de-trended and standardized weekly number of IS admissions during the whole study period. *p* values of the paired *t* tests, which compare the COVID-periods to their respective controls, are also presented. Dates of the most important restrictive and alleviative health emergency operative measures are marked in the timeline. sd, standard deviation; COVID-periods, periods of coronavirus disease 2019; IS, ischemic stroke

In the Wave-1 period, compared to the 1st control interval, we observed a significant decrease in the weekly IS admissions’ mean and median. In the control chart, during the Wave-1 period, a marked negative deviation from the trend could be observed: values below the − 2 SD control limit indicate alterations, and even if we consider the effect of multiple testing and use the − 3 SD control limit, the disruption in the trend is clearly visible. Paired *t* tests on IS admission z-scores also demonstrated a significant decline (Table 1, Figs. 2, 3, Table S1).

**Table 1:** Mean-based results of the statistical tests. This table shows the results of the *t* tests, which compared the mean values of the analyzed variables (raw number and z-score of IS admissions, IVTs and EVTs per week, and z-score of the ratio of de-trended weekly number of IVTs or EVTs and IS admissions) in the COVID-periods and the control intervals. Tests are paired where applicable (where the number of weeks equal). *SD* standard deviation, *IS* ischemic stroke, *IVT* intravenous thrombolysis, *EVT* endovascular therapy, *COVID-periods* periods of coronavirus disease 2019. Bold font indicates statistical significance

Variables	1st control period	Wave-1 period	*p* value	3rd control	Wave-1 period	*p* value	2nd control period	Wave-2 period	*p* value	3rd control period	Wave-2 period	*p* value
IS admissions (mean (SD))	2214.73 (225.85)	1225.67 (282.78)	** < 0.001**	1790.50 (167.27)	1225.67 (282.78)	** < 0.001**	2194.47 (352.19)	1314.00 (448.19)	** < 0.001**	1790.50 (167.27)	1314.00 (448.19)	**0.001**
z-score of IS admissions (mean (SD))	0.21 (0.86)	− 3.42 (1.08)	** < 0.001**	− 1.23 (0.64)	− 3.42 (1.08)	** < 0.001**	0.21 (1.34)	− 3.02 (1.69)	** < 0.001**	− 1.23 (0.64)	− 3.02 (1.69)	**0.001**
IVTs (mean (SD))	63.20 (7.18)	53.53 (10.70)	**0.001**	57.60 (7.97)	53.53 (10.70)	0.288	58.18 (10.57)	52.41 (8.40)	0.107	57.60 (7.97)	52.41 (8.40)	0.125
z-score of IVTs (mean (SD))	0.79 (0.85)	− 1.19 (1.26)	** < 0.001**	− 0.90 (0.96)	− 1.19 (1.26)	0.526	− 0.22 (1.25)	− 1.73 (1.00)	**0.002**	− 0.90 (0.96)	− 1.73 (1.00)	**0.045**
EVTs (mean (SD))	22.81 (5.25)	23.02 (4.28)	0.883	26.00 (4.08)	23.02 (4.28)	0.095	23.63 (5.03)	20.99 (4.89)	0.127	26.00 (4.08)	20.99 (4.89)	**0.009**
z-score of EVTs (mean (SD))	0.60 (1.29)	− 0.80 (0.98)	**0.001**	− 0.41 (1.05)	− 0.80 (0.98)	0.358	0.05 (1.23)	− 2.00 (1.25)	** < 0.001**	− 0.41 (1.05)	− 2.00 (1.25)	**0.002**
z-score of IVTs/IS admissions (mean (SD))	0.42 (0.86)	2.79 (1.95)	**0.001**	0.25 (1.23)	2.79 (1.95)	**0.001**	− 0.29 (1.03)	3.06 (5.94)	**0.028**	0.25 (1.23)	3.06 (5.94)	0.076
z-score of EVTs/IS admissions (mean (SD))	0.39 (1.31)	3.09 (1.84)	** < 0.001**	0.71 (1.49)	3.09 (1.84)	**0.002**	0.04 (1.59)	2.02 (4.23)	**0.045**	0.71 (1.49)	2.02 (4.23)	0.258

**Fig. 4:**
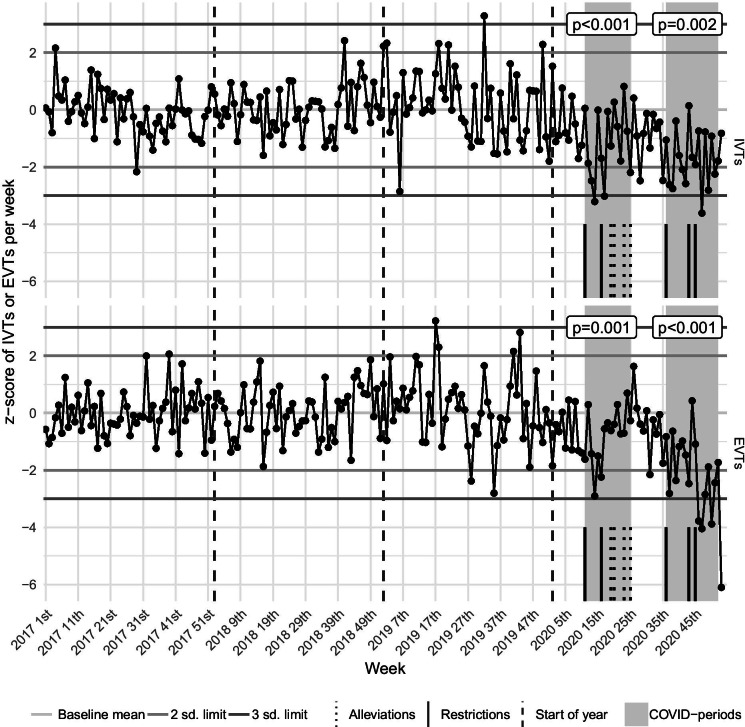
Control chart of IVTs and EVTs. These charts visualize the trend and changes using the de-trended and standardized weekly number of IVTs and EVTs during the whole study period. *p* values of the paired *t* tests, which compare the COVID-periods to their respective controls, are also presented. Dates of the most important restrictive and alleviative health emergency operative measures are marked in the timeline. sd, standard deviation; COVID-periods, periods of coronavirus disease 2019; IVT, intravenous thrombolysis; EVT, endovascular therapy

**Fig. 5:**
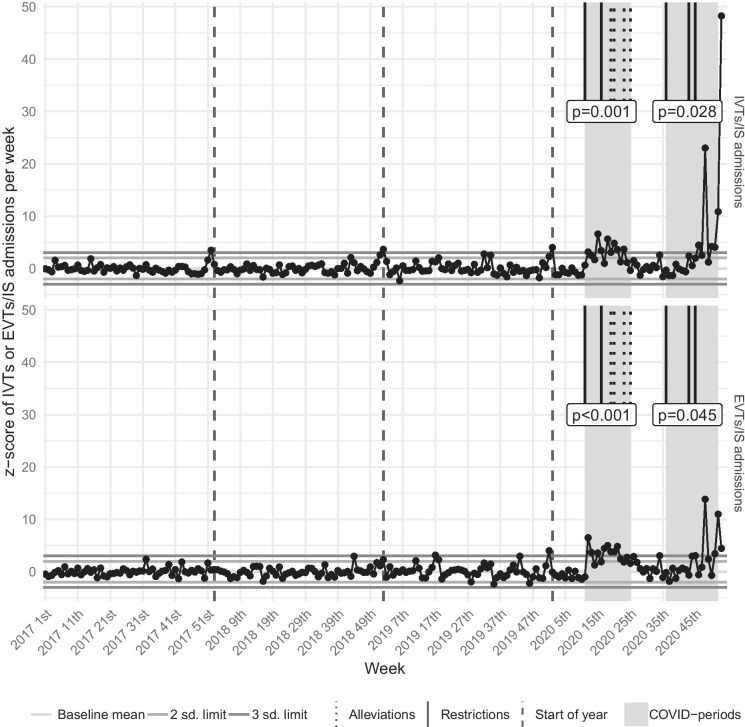
Control chart of the ratio of IVTs or EVTs and IS admissions. These charts visualize the trend and changes using the de-trended and standardized weekly number of IVTs or EVTs relative to IS admissions during the whole study period. *p* values of the paired *t* tests, which compare the COVID-periods to their respective controls, are also presented. Dates of the most important restrictive and alleviative health emergency operative measures are marked in the timeline. sd, standard deviation; COVID-periods, periods of coronavirus disease 2019; IVT, intravenous thrombolysis; EVT, endovascular therapy; IS, ischemic stroke

While the Wave-1 period did not alter the mean and median of weekly EVT numbers considerably, the weekly IVT numbers’ mean and median values reduced significantly in the Wave-1 period, compared to the 1st control interval (Table 1, Fig. 2, Table S1). Nevertheless, the de-trended and standardized weekly number of IVTs and EVTs showed a significant decrease in the Wave-1 period, representing a remarkable decline from the trend. In the control charts, the Wave-1 period’s effect on the weekly EVT numbers was milder but detectable and significant: several consecutive observations were below the centerline, there was a case of 2-out-of-3 consecutive weeks below the − 2 SD control limit, and the results of the difference tests on z-scores were also significant (Table 1, Fig. 4, Table S1). The trend analysis of the ratio of IVTs or EVTs and IS admissions showed a significant increase during the Wave-1 interval (Table 1, Fig. 5, Table S1). It implies that even though both the de-trended and standardized weekly number of IVTs, EVTs, and IS admissions reduced in the Wave-1 period, the decrease of IS admissions was disproportionally greater.

Compared to the Wave-1 period, in the 3rd control period, the weekly number of IS admissions showed a clearly detectable increase in the raw numbers and the de-trended and standardized data (Table 1, Fig. 3, Table S1). In contrast, compared to the Wave-1 period, the weekly number of IVTs and EVTs did not change significantly in the epidemic interlude, neither in the raw data nor in the de-trended and standardized data. Simultaneously, the ratio of IVTs or EVTs and IS admissions returned to the baseline levels. (Table 1, Fig. 4, 5, Table S1).

In the Wave-2 period, compared to the 2nd control interval, the weekly IS admissions’ mean and median values significantly decreased, but the mean and median of weekly IVTs and EVTs did not show a remarkable change (Table 1, Fig. 2, Table S1). However, the de-trended and standardized data analysis demonstrated a significant drop from the trend of IS admissions, IVTs, and EVTs. In the control charts, during the Wave-2 period, the ratio of IVTs or EVTs and IS admissions significantly increased, reaching even more extreme values (values beyond the 10 SD limit) than in the Wave-1 interval (Table 1, Figs. 3, 4, 5, Table S1).

Comparing the raw numbers and z-scores of IS admissions and reperfusion interventions from the Wave-2 period with the 3rd control period, we found similar results as compared with the 2nd control period, with two exceptions: compared to the epidemic interlude, not only the z-scores of EVT reduced significantly in the Wave-2 period, but also the mean and median of raw numbers. Although the ratio of IVTs or EVTs and IS admissions showed an extreme increase in the control charts, the mean and median of z-scores did not alter significantly. The cause of this apparent contradiction is that the mean and median of z-scores use the whole length of the Wave-2 period, but the analyzed ratios’ z-scores started to increase significantly only at the 43rd week of 2020 (Table 1, Figs. 3, 4, 5, Table S1).

### General analysis of the control charts

In the IS admissions’ control chart, the weekly number of IS admissions shows mild seasonality, guided mainly by vacations and national holidays. These changes may have inflated the variance in the baseline period (Fig. 3). In the control charts of reperfusion interventions, the baseline periods do not show any striking artifacts, only a mild increase in the variance can be seen. Occasional random extremes (“false alarms”) occurred as expected (Fig. 4).

The winter holiday season (generally the 51st–1st weeks of a calendar year) tends to bring the weekly number of IS admissions extremely low (below a distance of -3 SD), while the weekly IVT and EVT numbers do not alter remarkably. Thus concurrently, the ratio of IVTs or EVTs and IS admissions shows a significant (above a distance of 2 or 3 SD) increase (Figs. 3, 4, 5). The summer holiday season (generally the 24th–35th weeks of a calendar year) has a similar but longer-lasting and less potent effect on the weekly number of IS admissions, IVTs, and EVTs (Figs. 3, 4, 5).

### Linear regression analysis

**Fig. 6:**
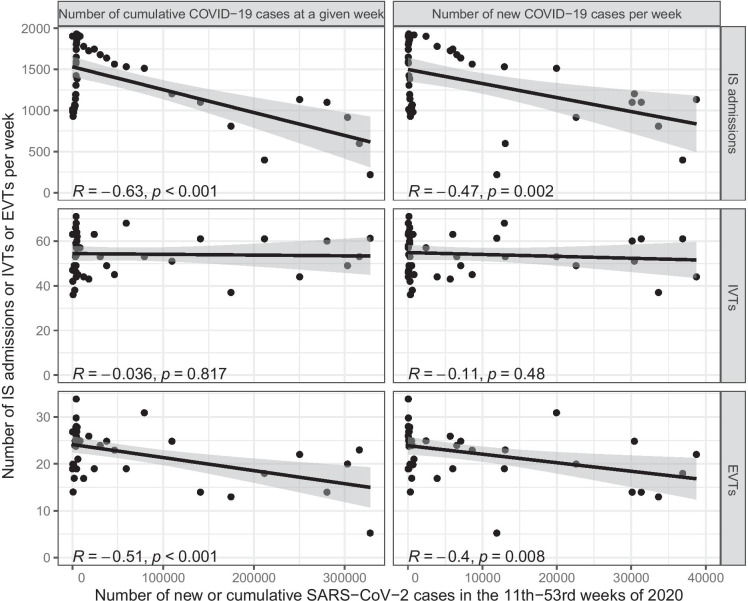
Relationship between the weekly number of IS admissions, IVTs, and EVTs, and the new or cumulative SARS-CoV-2 cases per week in Hungary. This figure visualizes the linear regression analysis results, which compared the weekly number of IS admissions, IVTs, and EVTs with the new or cumulative SARS-CoV-2 infections’ weekly number in Hungary during the comprehensive interval of the 11th–53rd weeks of 2020, which extends from the start of the Wave-1 period (representative of the first COVID-19 epidemic wave in Hungary) to the end of 2020. R: Pearson’s correlation coefficient; *p*, *p* value of the correlation (same as the *p* value of the linear regression); grey area, 95% confidence interval of the slope; IS, ischemic stroke; IVT, intravenous thrombolysis; EVT, endovascular therapy; SARS-CoV-2, severe acute respiratory syndrome coronavirus 2; COVID-19, coronavirus disease 2019

The weekly number of IS admissions, IVTs, and EVTs was compared with the new or cumulative SARS-CoV-2 infections’ weekly number in Hungary during the comprehensive interval of the 11th–53rd weeks of 2020. The weekly number of IS admissions and EVTs significantly decreased with the increase of the new or cumulative COVID-19 cases per week (negative linear correlation), while the number of IVTs did not show a significant linear correlation with the number of SARS-CoV-2 infections (Fig. 6). The relationship between variables may not be linear in several cases, but we did not investigate this angle any further as this sub-analysis was mainly exploratory and just a complementary tool.

## Discussion

Our study revealed that the mean and median values of weekly IS admissions and reperfusion interventions showed a decrease only in some measure during the two epidemic waves of COVID-19 in Hungary. However, the control charts demonstrated that these values reflect a significant disruption in the trends and decline in the number of IS admissions, IVTs, and EVTs. It is also notable that results regarding IS admissions were similar if we computed the number of IS admissions as the number of cases where ICD I63 or I64 or I66 codes presented only as primary discharge diagnosis (i.e., main diagnosis for admission) in the reimbursement database of NHIFH (data presented in the online supplementary Fig. S1-2).

Notwithstanding that a negative deviation from the trend could be observed both in IS admission numbers and in IVT and EVT numbers, the decline’s dynamic and amplitude have differed for each variable. During the COVID-periods, the number of IS admissions showed a high amplitude negative steep wave of decrease with a significant restoration in the epidemic interlude. Similarly, the EVT numbers decline also presented with a negative wave dynamic but with a smaller amplitude and without significant rearrangement in the epidemic interlude. In contrast, the IVT’s decline from the trend was rather stepwise (larger amplitude but less dynamic) during the first wave of the COVID-19 outbreak, and the extent of the deviation from the trend persisted through the epidemic interlude and the second epidemic wave. Additionally, our study demonstrated a significant negative correlation between the number of SARS-CoV-2 cases in Hungary and the number of IS admissions and EVTs. However, the number of IVTs changed regardless of the amplitude of the COVID-19 epidemic waves.

These results suggest that multiple different factors might play a role in disrupting the trends of the analyzed characteristics. Our study is unable to evaluate the causes, but several factors can be hypothesized. Some authors suggest that stroke incidence might decrease, but the European Stroke Organization and most authors agree that there is no well-grounded reason to assume that stroke incidence declined since the onset of the COVID-19 crisis [17, 23, 34, 59]. Furthermore, an overwhelmed emergency medical system and broken chain of stroke, prehospital and institutional epidemiological precautionary measures, stringent adherence to revascularization therapies’ guidelines, social distancing, fear of acquiring the SARS-CoV-2 infection, and other changes in the social behavior also could be hypothesized [14, 16, 18, 20, 23, 24, 28], 31, [31, 33, 34, 60–62]. We speculate that the health emergency operative measures as a 20–60% reduction in the number of available hospital beds to ensure the care of COVID-19 patients; a complete or partial restriction of elective health care could also play a role [10, 46, 47, 49–53].

During the two epidemic waves, the number of IS admissions decreased to a disproportionally larger extent than the number of reperfusion interventions. We hypothesize that the health emergency operative measures could be one of the causes because the dynamic of the disproportionally greater decrease of IS admissions seems to be mostly concurrent with the health emergency operative measures. During the Wave-1 period, the peak of the disproportionally greater reduction of IS admissions overlaps when 50–60% of beds had to be reserved for COVID-19 patients, and the gradual termination of this phenomenon co-occur with the four-step alleviation of restrictive measures. At the Wave-2 period, the IS admission numbers started to decrease to a disproportionally larger extent when the COVID-designated hospitals’ number was expanded, and 20–40% of beds had to be reserved for COVID-19 patients. It could also be presumed the COVID-19 itself or the lifestyle altered by confinement measures could change the proportion of IS patients eligible for reperfusion therapies. Besides, changes in patients’ social behavior (non-disabling IS cases, where reperfusion interventions would not have been indicated, could stay at home because of the fear of getting infected) could also contribute. Since stroke mimics are usually characterized by mild neurological symptoms, similarly to non-disabled IS cases, their reduced presentation, led by the fear of getting infected, might also be presumed in the COVID-periods [63]. However, while it could contribute to the decrease of IVTs, it cannot explain the decline of IS admissions because stroke mimics are not included in our cohort of IS admissions.

In the COVID-periods, a numerical pattern very similar to the winter and summer holiday seasons could be observed (an increase in the ratio of IVTs or EVTs and IS admissions), which might indirectly support both the behavioral and the healthy policy hypothesis. One might hypothesize that patients may not seek medical attention for acute neurological symptoms during winter and summer holidays unless they perceive that the symptoms are so severe (potentially IVT and EVT candidate cases) that it allows them no other choice. This theory could explain the increase in the ratio of reperfusion interventions and IS admissions during the winter and summer holiday seasons. During the epidemic waves, patients might have a very similar attitude, which might be motivated by the fear of getting infected, rather than not wanting to miss festivities. In the Wave-1 period, when patients might have little idea of what to expect from the COVID-19 epidemic, the fear might be high, which could cause an abrupt increase in the ratio of reperfusion interventions and IS admissions. While in the Wave-2 interval, when patients already had experiences of the epidemic’s dangers, the fear might not reach Wave-1 level, despite SARS-CoV-2 cases being higher, until the COVID-19 case numbers and deaths reached an exceptionally high value. This theory could explain the later onset increase in the ratio of reperfusion interventions and IS admissions in the Wave-2 period. It is also presumable that non-acute IS admissions for follow-up investigations are less likely in the winter and summer holiday seasons, which might also explain the increase in the ratio of reperfusion interventions and IS admissions in these periods. In the Wave-1 interval, when governments and healthcare authorities might have little knowledge about the COVID-19 epidemic, strict restrictive measures were abruptly implemented. These restrictive measures rapidly made it impossible to admit elective, non-acute IS cases, which might also increase the ratio of reperfusion interventions and IS admissions in the Wave-1 interval. Presumably based on the experiences from the Wave-1, despite the higher COVID-19 case numbers, strict confinement measures and restriction of elective health care took place later in the Wave-2 period, which might also contribute to the later onset increase in the ratio of reperfusion interventions and IS admissions. Additionally, it can also be presumed that during the COVID-19 epidemic, patients were less likely to accept elective hospital admissions because of the fear of getting infected, even if it was allowed.

Behind the decline of EVT numbers, deceleration of the continuous growth of EVT numbers could also be hypothesized, which overlaps the time of the COVID-19 epidemic. However, it seems less likely since in our database annually maximum of 3% of patients with ICD-10 I63/I64/I66 primary discharge diagnosis codes were treated with EVT, while population-based studies from 2016 to 2017 estimated that 7–16% of all IS admissions are potentially eligible for EVT [64–66]. Moreover, evidence from recent years might increase further the proportion of acute IS patients eligible for EVT, making the theory of EVT’s deceleration less probable [2–9]. Additionally, the inclusion of the first 10 weeks of 2020 in the baseline period also makes such a steep and prominent deviation from the EVT’s baseline less like solely due to the deceleration of EVT’s growth.

Our study’s main limitations are our database’s reimbursement purpose and our research’s retrospective and observational nature and the different coding practices that had to be addressed. Furthermore, our study does not cover the entire second wave of the COVID-19 epidemic in Hungary.

In conclusion, our study demonstrated a significant disruption in IS care during the COVID-19 epidemic in Hungary. In the negative impact of the COVID-19 epidemic on IS care, multiple different factors might play a role. Furthermore, we revealed that the number of IS admissions decreased to a disproportionally larger extent than the number of reperfusion interventions during the first and second waves of the COVID-19 outbreak in Hungary, which could partially be explained by the effect of health emergency operative measures and changes in patients’ social behavior. Further studies are needed to evaluate the causes of our observations. Our study highlights the importance of the IS care system’s continuous surveillance and what we learned from these two COVID-19 waves we can use to preserve IS care in subsequent waves or future epidemics.

## Supplementary Information

Below is the link to the electronic supplementary material.

Supplementary file1 Changes in the raw weekly number of primary discharge diagnoses during the COVID-periods. This figure shows the raw weekly number of primary discharge diagnoses with ICD-10 I63/64/66 codes in the COVID-periods and their respective controls using standard box plots. p-values of the paired Wilcoxon-Man-Whitney tests, which compare the COVID-periods to their respective controls, are also presented. full dots: Tukey-defined outliers; p-value: *** p<0.001, **** p<0.0001; COVID-periods, periods of coronavirus disease 2019; ICD-10, 10th version of The International Statistical Classification of Diseases and Related Health (PDF 5 KB)

Supplementary file2 Control chart of primary discharge diagnoses. This graph visualizes the trend and changes using the de-trended and standardized weekly number of primary discharge diagnoses with ICD I63/64/66 codes during the whole study period. p-values of the paired t-tests, which compare the COVID-periods to their respective controls, are also presented. Dates of the most important restrictive and alleviative health emergency operative measures are marked in the timeline. sd, standard deviation; COVID-periods, periods of coronavirus disease 2019; ICD-10, 10th version of The International Statistical Classification of Diseases and Related Health (PDF 23 KB)

Supplementary file3 Median-based results of the statistical tests. This table shows the results of the Wilcoxon–Mann–Whitney tests, which compared the median values of the analyzed variables (raw number and z-score of IS admissions, IVTs and EVTs per week, and z-score of the ratio of de-trended weekly number of IVTs or EVTs and IS admissions) in the COVID-periods and the control intervals. Tests are paired where applicable (where the number of weeks equal). IQR, interquartile range; IS, ischemic stroke; IVT, intravenous thrombolysis; EVT, endovascular therapy; COVID-periods, periods of coronavirus disease 2019. Bold font indicates statistical significance (XLSX 12.9 KB)

## Data Availability

The data of this study are available from the corresponding author upon reasonable request.

## Code availability

R version 4.0.3 is a free software.
