# Supplementary figures and images for: Impact of COVID-19 on ischemic stroke care in Hungary
Source: GeroScience. 2021 Aug 18;43(5):2231–48. doi: 10.1007/s11357-021-00424-z (PMC8371604; doi:10.1007/s11357-021-00424-z)

1st control and Wave-1

2nd control and Wave-2

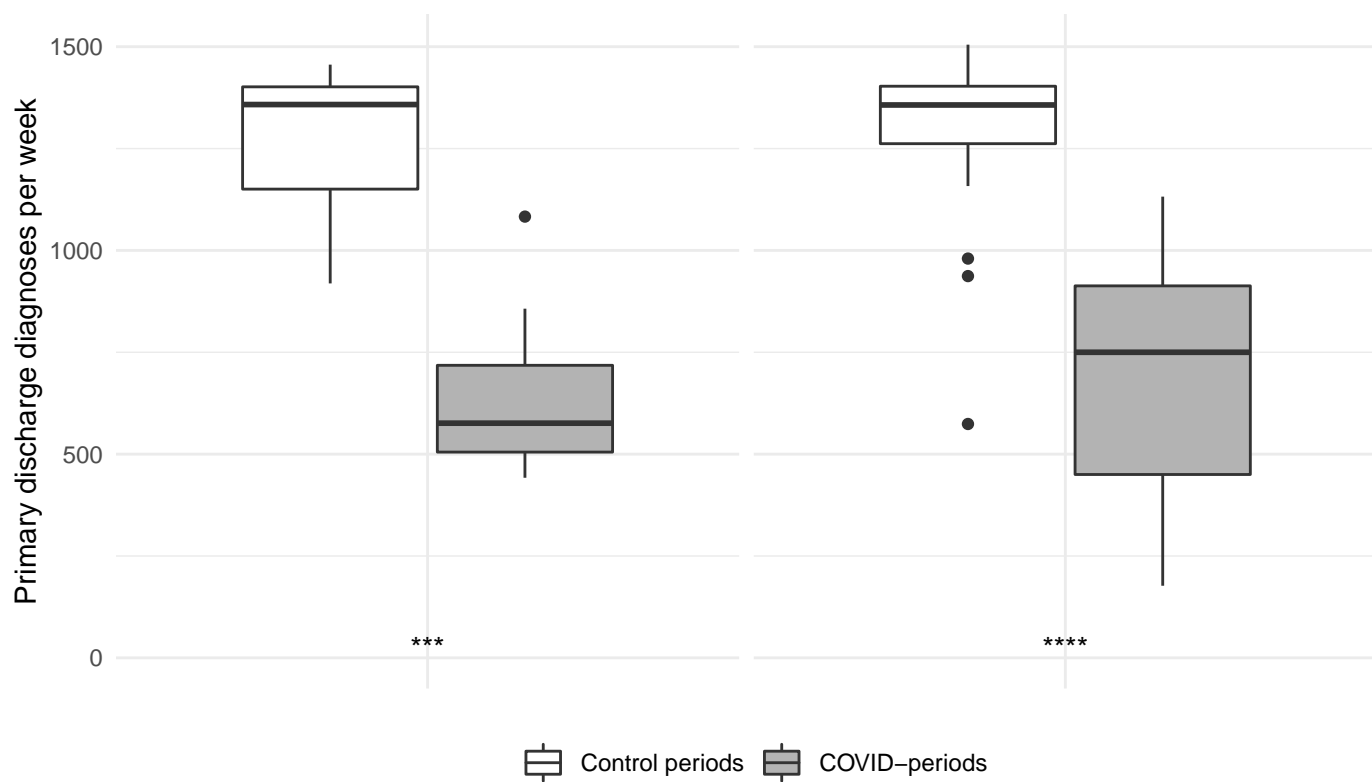

Supplement: Supplementary file 1 — Supplementary file1 Changes in the raw weekly number of primary discharge diagnoses during the COVID-periods. This figure shows the raw weekly number of primary discharge diagnoses with ICD-10 I63/64/66 codes in the COVID-periods and their respective controls using standard box plots. p-values of the paired Wilcoxon-Man-Whitney tests, which compare the COVID-periods to their respective controls, are also presented. full dots: Tukey-defined outliers; p-value: *** p<0.001, **** p<0.0001; COVID-periods, periods of coronavirus disease 2019; ICD-10, 10th version of The International Statistical Classification of Diseases and Related Health (PDF 5 KB) [file 11357_2021_424_MOESM1_ESM.pdf]

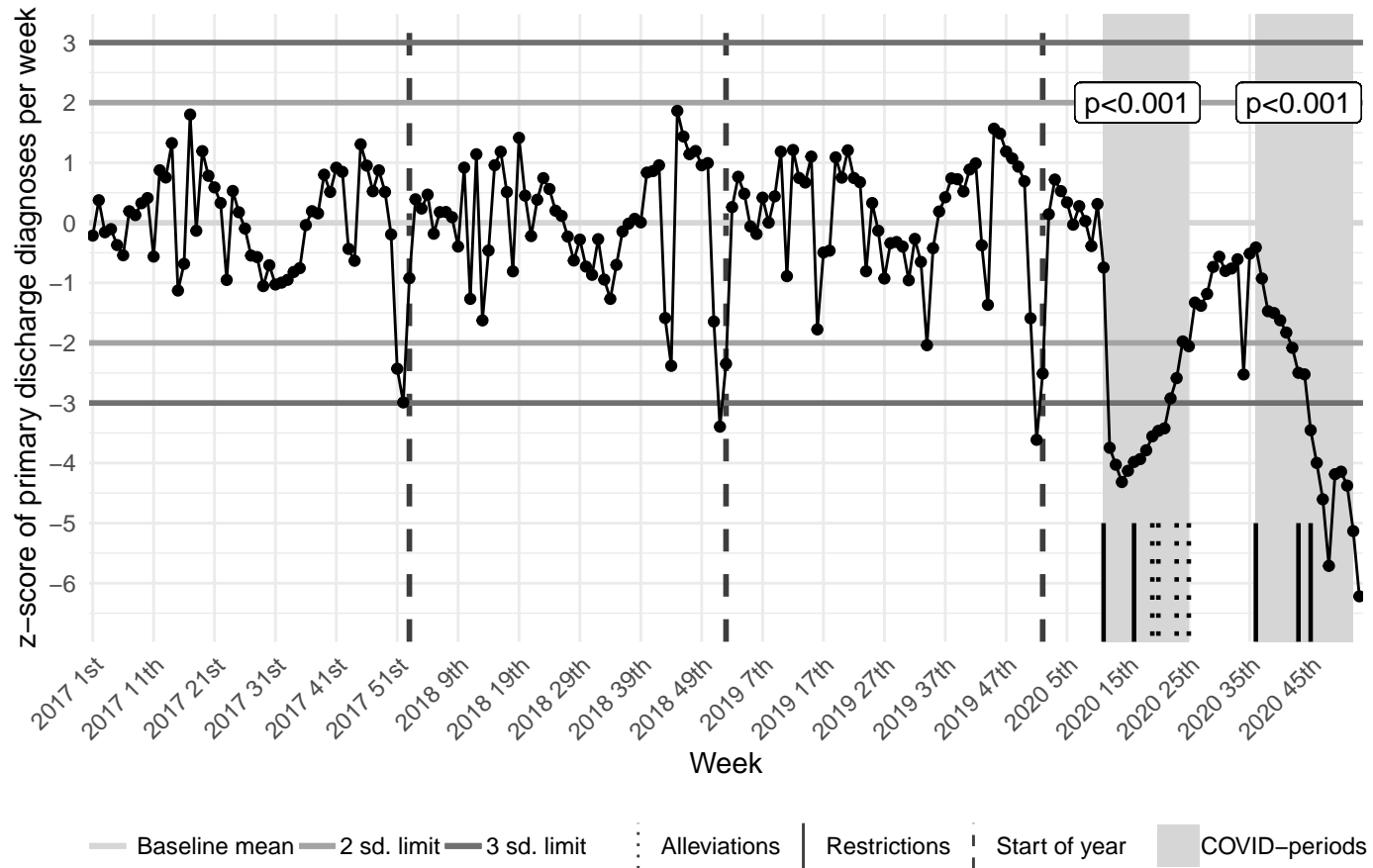

Supplement: Supplementary file 2 — Supplementary file2 Control chart of primary discharge diagnoses. This graph visualizes the trend and changes using the de-trended and standardized weekly number of primary discharge diagnoses with ICD I63/64/66 codes during the whole study period. p-values of the paired t-tests, which compare the COVID-periods to their respective controls, are also presented. Dates of the most important restrictive and alleviative health emergency operative measures are marked in the timeline. sd, standard deviation; COVID-periods, periods of coronavirus disease 2019; ICD-10, 10th version of The International Statistical Classification of Diseases and Related Health (PDF 23 KB) [file 11357_2021_424_MOESM2_ESM.pdf]
